# An economic model to understand the cost-effectiveness of olanzapine orally dispersible tablets (ODT) and olanzapine film coated tablets as a group compared with other oral atypical antipsychotics for treating schizophrenia in Morocco

**DOI:** 10.1186/s12991-024-00516-y

**Published:** 2024-09-18

**Authors:** Ahmed Tazi, Faouzi Errachidi, Dipesh Sonawane, Ghizlane Tahri, Sameer Rao, Suyog Mehta

**Affiliations:** 1 Department of Pharmacology & Biology, Sidi Mohamed Ben Abdellah University (USMBA), Fez, Morocco; 2 Department of Medical Affairs and Clinical Research, Sun Pharmaceutical Industries Limited, Western Express Highway, Goregaon East (Near JVLR Junction), Mumbai, 400 063 India; 3 Department of Medical Affairs and Clinical Research, Sun Pharmaceuticals Morocco LLC, El Maârif-Casablanca, Morocco; 4 Department of Medical Affairs and Clinical Research, Sun Pharma Laboratories Ltd, Mumbai, India

**Keywords:** Orally dispersible tablets (ODT), Standard oral tablet (SOT), Olanzapine, Schizophrenia, Cost-effective

## Abstract

**Background:**

Antipsychotic medications are the primary treatment for schizophrenia, with olanzapine being an effective medication for schizophrenia. The economic cost for each individual with schizophrenia is high, with antipsychotic medication being a major expense. This study aims to develop an economic decision model that compares different treatment options for schizophrenia patients, including olanzapine Orally Dispersible Tablets (ODT), olanzapine [ODT + Standard Oral Tablet (SOT)], risperidone (ODT + SOT), and aripiprazole (ODT + SOT), to determine their cost-effectiveness with an objective to optimize healthcare resource allocation in Morocco.

**Methods:**

The study used published medical literature and a clinical expert panel to develop a decision analytic model. This model was designed to capture parameters such as adherence levels, treatment discontinuation, relapse with and without hospitalization, quality-adjusted life years (QALYs), treatment-related adverse events, healthcare resource utilization, and associated costs. The main outcomes of interest included the total annual direct cost per treatment, QALYs, and incremental cost-effectiveness ratio (ICER) per 1 QALY gained. One-way and probabilistic sensitivity analyses were employed to account for parameter uncertainty.

**Results:**

According to the simulation model, the ODT and ODT + SOT as a group form of olanzapine was the most effective treatment option in terms of the lowest percentages of inpatient relapse, and patients who remained stable (11% and 79% respectively) than risperidone (19% and 62% respectively) and aripiprazole ODT (26% and 50% respectively) and ODT + SOT formulation groups. Olanzapine (ODT + SOT) therapy group was cost-effective when compared to the combined group of ODT + SOT forms of risperidone [ICER: Moroccan Dirham (MAD) 103,907], and aripiprazole (ICER: MAD 65,047). Additionally, olanzapine ODT was found to be cost-effective compared to olanzapine SOT with an ICER of MAD 3921, risperidone ODT with an ICER of MAD 1,02,298, risperidone SOT with an ICER of MAD 31,088, and aripiprazole ODT or SOT formulations. All the above ICERs fall under the willingness-to-pay threshold in Morocco of MAD 250,832.40. Sensitivity analyses confirmed the reliability of the findings.

**Conclusions:**

The model concluded that olanzapine ODT is the most cost-effective first-line treatment option for schizophrenia in Morocco when compared to other atypical antipsychotic medications in ODT and SOT formulations.

## Introduction

Approximately 24 million individuals globally are affected by schizophrenia, a severe and persistent mental disorder [1], with an incidence of 1 to 3 per 10,000 individuals and a prevalence of 10 to 100 per 10,000 individuals [2]. The disease imposes substantial health, social, and financial burdens on individuals, their families, caregivers, and the community [3–6]. The impact of diseases and medical interventions on populations are assessed using two key metrics—Quality Adjusted Life Years (QALYs) and Years of Healthy Life Lost due to Disability (YLDs) [7]. QALYs is a measure that integrates life duration and life quality [8]. It evaluates the worth of medical interventions by taking into account both the improvement in life expectancy and the prolongation of life itself. One QALY is equivalent to a year of good health [9]. QALYs enable the comparison of the efficacy of various therapies across a range of diseases and conditions by aggregating health outcomes into a single metric [10]. The years of healthy life lost as a result of having a disability or medical condition are represented by YLDs. It quantifies the burden of disease that causes disability rather than death. The number of incident cases multiplied by the average illness duration plus a weight factor representing the severity of the health loss determines the YLDs. YLDs offer a comprehensive picture of how various health conditions affect population well-being over time by capturing the non-fatal impact of diseases [11, 12]. Patients between the ages of 25 and 54 experience the highest burden of the disease [13], which accounts for 1.7% of all years of healthy life lost due to disability worldwide in 2016 [14]. Schizophrenia’s lifetime prevalence in 12 African countries ranges from 1 to 4.4% [15], while 1 in 7 children and adolescents in sub-Saharan Africa experience psychological difficulties [16]. In Morocco, an estimated 92,573 people live with schizophrenia, with a burden measured in disability-adjusted life years (DALYs) per 100,000 at 155.29 [17], although recent epidemiological statistics are unavailable due to the lack of a government registry. DALYs represent the total number of years lost due to illness, disability, or premature death, combining both the years of life lost (YLL) and the years lived with disability (YLD).

Antipsychotic medications are the primary treatment for schizophrenia [18], with first and second-generation antipsychotics being the two main groups [19]. Olanzapine is an effective medication for schizophrenia and bipolar disorder [20]. Newer antipsychotic formulations have been developed to increase efficacy and ease of administration. Olanzapine is available in orally disintegrating tablets (ODTs) and standard oral tablets (SOTs), with ODTs offering an alternative for patients who are unable or unwilling to swallow pills [21]. Studies indicate that olanzapine ODT can enhance compliance and reduce the likelihood of relapse and hospitalization, which could result in greater cost-effectiveness [22]. Patients with schizophrenia who do not adhere to their oral medication regimen are at risk of relapse and hospitalization [23]. To improve adherence, long-acting formulations of antipsychotic medications have been developed. These formulations aim to ensure patients’ long-term compliance with their treatment regimen and achieve better patient outcomes [24].

Schizophrenia places a significant financial burden on the healthcare system and society [25]. The economic cost for each individual with schizophrenia is high, with antipsychotic medication being a major expense [13]. Additionally, compared to Europe (12.5%), out-of-pocket costs for child mental health services in African nations are considerably higher (71.4%) [26]. There is a need to determine the economic burden of schizophrenia in Morocco and evaluate the cost-effectiveness of different treatment options to optimize healthcare resource allocation. According to published economic analyses, atypical (second-generation) antipsychotics are more affordable than traditional (first-generation) treatment [27]. Recent studies have demonstrated that the ODT formulation is linked to greater patient preference, convenient administration, and higher adherence rates, all of which may lessen the treatment burden on both patients and caregivers [28].

The primary objective of the study was to develop an economic decision model that compared different treatment options for schizophrenia patients in Morocco, including olanzapine ODT, olanzapine (ODT + SOT), risperidone (ODT + SOT), and aripiprazole (ODT + SOT). The study evaluated the relative clinical benefits and associated costs of these options to determine their cost-effectiveness. These study results may provide valuable information for clinicians and policymakers in Morocco regarding the most cost-effective treatment options for schizophrenia. Ultimately, we believe that the findings from this study have the potential to improve patient outcomes while optimizing the allocation of healthcare resources in the country.

## Methods

### Model overview

A model for decision analysis was created to assess the cost-effectiveness of olanzapine ODT versus SOT. The structure of the model used in the decision analysis represented the pathway of a patient with schizophrenia receiving either the ODT and SOT formulations of olanzapine, risperidone, and aripiprazole. Patients were classified as compliant, partially compliant, or non-compliant with the treatment, and their adherence level was taken as the determinant as to whether patients may either remain stable or have a relapse, which may or may not necessitate hospitalization.

**Fig. 1:**
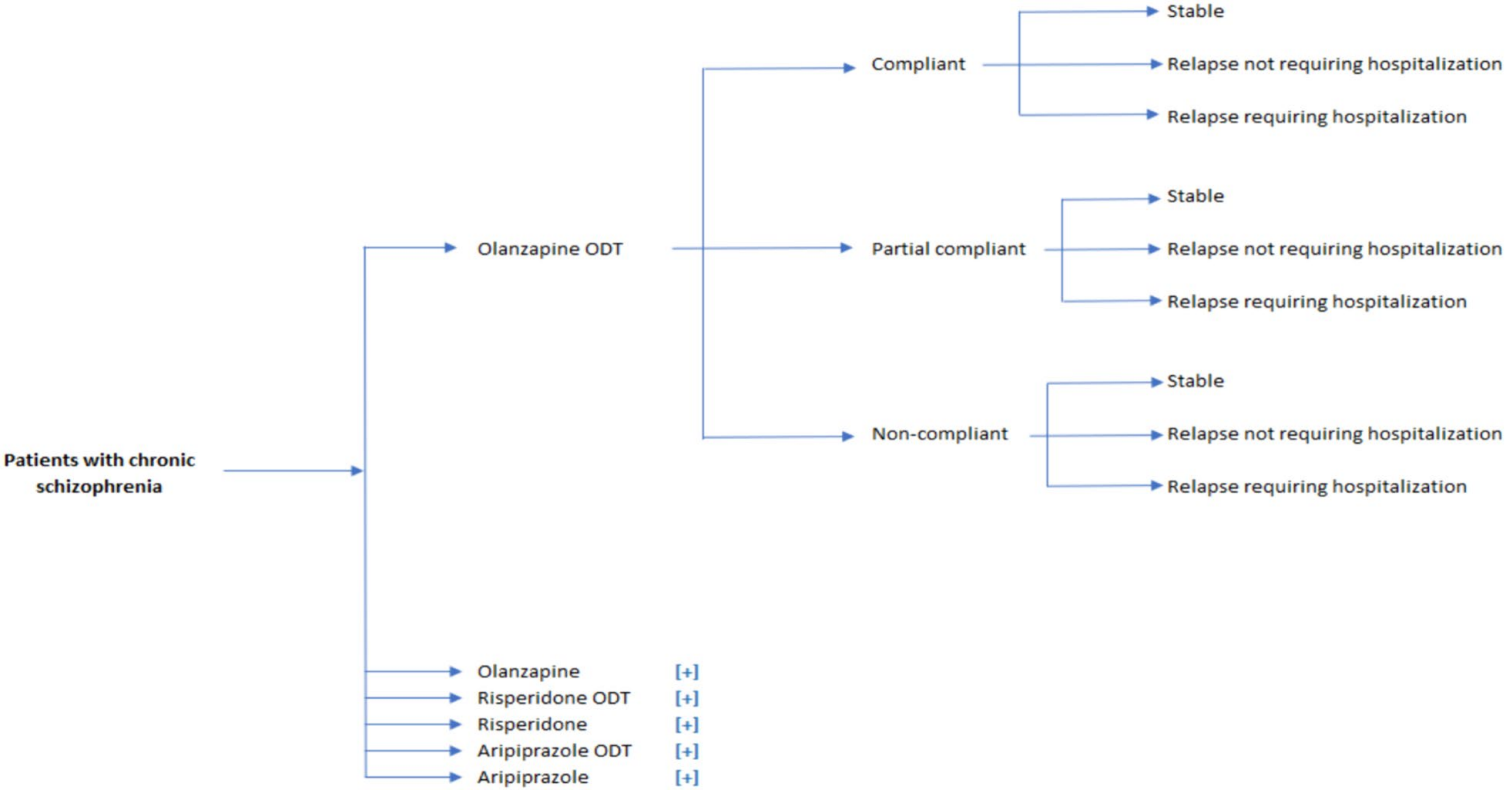
Illustration of the model’s framework

The model used in the decision analysis included six treatment groups that involved three commonly used atypical antipsychotics, including olanzapine, risperidone, and aripiprazole, that were included in both ODT and SOT formulations in the model. Additionally, the model incorporated three treatment groups that included ODT and SOT formulations of different antipsychotics as a group [risperidone (ODT + SOT), aripiprazole (ODT + SOT), and olanzapine (ODT + SOT)]. The model was designed to simulate the standard of care process for schizophrenia patients for a period of one year, taking into account the dynamic nature of their condition. The model considered several input parameters, such as adherence rates, relapse with or without hospitalization, health state utilities, adverse events, resource utilization in the healthcare system, and direct healthcare costs. The simulation involved 1,000,000 patients and led to the prediction of significant clinical outcomes such as quality-adjusted life years. As the model was developed for Morocco, all Costs are denoted in Moroccan Dirhams (MAD) using 2022 values. Additionally, based on the cost data received from Morocco, ODT formulations of antipsychotic medications were more expensive than their SOT counterparts. The approach used in the model was intent-to-treat, where all direct medical costs estimated were attributed to the patient’s initial treatment received. Additionally, adverse reactions that may be reported by patients during treatment, such as extrapyramidal symptoms (EPS), significant weight gain, or diabetes we also accounted for, in the model. Figure 1 presents an overview of the model.

### Key clinical and economic input values

Given a lack of available clinical data estimates for Morocco, we had to use data published in a cost effectiveness study by Ascher-Svanum et al. [22] to obtain values for key clinical endpoints. In cases where data could not be obtained from peer-reviewed articles, expert opinion of psychiatrists in Morocco was utilized to understand treatment patterns and resource utilization. The model assumed that ODT formulations of the three antipsychotics assessed were equal in efficacy and safety to their respective SOT formulations based on all clinical input parameters, with the exception of greater adherence in patients receiving ODT which was in line with published comparative data.

### Adherence levels

As there is no publicly available or published data on adherence rates, we used the following data from published literature as input parameters in the model. Consistent with prior research, adherence levels were categorized based on the medication possession ratio (MPR) as: adherent (MPR ≥ 80%), partially adherent (MPR ≥ 60%, ≤ 80%), and non-adherent (MPR ≤ 60%). The adherence rates used in the model are shown in Table 1 along with the data source.

**Table 1:** Adherence rates by medication

Medication	Compliant (%)	Partially compliant (%)	Non-compliant (%)	Source
Olanzapine	23	43	34	Ascher-Svanum et al. (US study) [22]
Risperidone	21	39	40	
Aripiprazole	19	35	46	
ODT olanzapine	37	29	34	
ODT risperidone	35	25	40	
ODT aripiprazole	33	21	46	

*ODT* orally disintegrating tablet (formulation)

### Relapse rates

Table 2 outlines the assumptions used in the study for the probability of the first relapse that requires hospitalization and relapse not requiring hospitalizations by adherence category for each medication.

**Table 2:** Relapse rates requiring and not requiring hospitalization

Medication	Compliant (%)	Partial compliant (%)	Non-compliant (%)	Source
Relapse rates requiring hospitalizations				
Olanzapine	2	4	5	Ascher-Svanum et al. (US study) [22]
Risperidone	4	6	9	
Aripiprazole	5	9	12	
ODT olanzapine	2	4	5	
ODT risperidone	4	6	9	
ODT aripiprazole	5	9	12	
Relapse rates not requiring hospitalizations				
Olanzapine	2	3	5	Ascher-Svanum et al. (US study) [22]
Risperidone	4	6	9	
Aripiprazole	5	8	11	
ODT olanzapine	2	3	5	
ODT risperidone	4	6	9	
ODT aripiprazole	5	8	11	

*ODT* orally disintegrating tablet (formulation)

### Treatment-emergent adverse events

To simulate the effects of antipsychotic treatment, the model requires assumptions about the likelihood of patients experiencing various types of treatment-related adverse events, such as extrapyramidal symptoms (EPS), clinically significant weight gain (defined as an increase in weight of at least 7% from baseline weight), and diabetes. Table 3 outlines the initial assumptions about treatment-related adverse events for each medication and their data sources.

**Table 3:** Treatment emergent adverse event

Medication	Adverse events			Source
	EPS (%)	Bodyweight gain (%)	Any other (diabetes) (%)	
Olanzapine	16	30	3	Ascher-Svanum et al. (US study) [22]
Risperidone	25	14	3	
Aripiprazole	21	7	2	
Olanzapine ODT	16	30	3	
Risperidone ODT	25	14	3	
Aripiprazole ODT	21	7	2	

*EPS* extrapyramidal symptoms, *ODT* orally disintegrating tablet (formulation)

### Utility and quality-adjusted life years

The model’s starting utility values for the nine potential scenarios involving levels of adherence and relapse status are presented in Table 4.

**Table 4:** Utility values for health states

Medical condition	Compliance			Source
	Compliant	Partially compliant	Non-compliant	
Stable	0.88	0.75	0.75	Ascher-Svanum et al. (US study) [22]
Relapse not requiring hospitalization	0.74	0.63	0.63	
Relapse requiring hospitalization	0.53	0.53	0.42	

### Medication cost

Daily dose amounts and medication expense are frequently correlated. We used the daily dose levels reported by psychiatrists in Morocco to ensure that schizophrenia patients receive equivalent medication dosages. Table 5 shows the 2022 net wholesale price, which reflects the baseline model assumptions for dosing and expense for each drug. It demonstrates that ODT antipsychotics are more expensive in Morocco as compared to SOT equivalents.

**Table 5:** Economic input parameters; medication costs

Medication	Cost	Source
Olanzapine	MAD 9.7	Morocco study
Risperidone	MAD 9.6	
Aripiprazole	MAD 10.6	
Olanzapine ODT	MAD 11	
Risperidone ODT	MAD 19.4	
Aripiprazole ODT	MAD 13.7	

*ODT* orally disintegrating tablet (formulation)

### Healthcare resource utilization

Table 6 provides information on the assumptions made regarding the utilization of eight different types of medical services for 5 different patient results, along with the sources of data used to generate this information.

**Table 6:** Healthcare resource utilization

Resources	Stable days (per patient per month)	Relapse rates (not requiring hospitalization) per event	Relapse rates (requiring hospitalization) per event	EPS per event	Body weight gain per event	Source
Hospitalisation	0	0	11.7	0	0	Ascher-Svanum et al. (US study) [22]
Ambulatory care centre	0	1.25	1.25	0	0	
Emergency department	0	1	1	0	0	
Doctor visits	1	1	1	1	0.5	
Psychiatric clinic visits	1.5	2	2	1	2.5	
Hours of home care	0	2.75	2.75	0	0	
Hours of group counselling	0.5	1.5	1.5	0	5	
Nutritionist visits	0	0	0	0	2.5	

*EPS* extrapyramidal symptoms, *ODT* orally disintegrating tablet (formulation)

### Healthcare resource cost

Table 7 presents the initial expenses of each healthcare resource used. The costs of each unit were adjusted for inflation to reflect the value of the Moroccan MAD in 2022, employing the section of the consumer price index that pertains to healthcare services.

**Table 7:** Healthcare resource cost

Resources	Cost/unit	Source
Hospitalisation	MAD 1058.30	Morocco study
Ambulatory care centre	MAD 5220.42	Ascher-Svanum et al. (US study) [22]
Emergency department	MAD 550.00	Morocco study
Any other: length of stay per admission	MAD 265.00	Morocco study
Outpatient care		
Doctor visits	MAD 571	Morocco study
Psychiatric clinic visits	MAD 550	Morocco study
Hours of home care	MAD 854	Ascher-Svanum et al. (US study) [22]
Hours of group counselling	MAD 740	Ascher-Svanum et al. (US study) [22]
Nutritionist visits	MAD 250	Morocco study
Any other: medication	MAD 229	Morocco study

### Model outcome measures

#### Clinical outcomes

The model estimates three critical clinical outcomes: the percentage of patients who experience an outpatient relapse, those who experience an inpatient relapse, and those who do not experience either an outpatient relapse or an inpatient relapse (i.e., stable).

#### Economic outcomes

The model reports mean total direct healthcare costs for ODT formulation along with ODT plus SOT formulation as a group for the all three antipsychotic drugs.

#### Cost-effectiveness information

The cost per one QALY gained for each medication is the main measure of cost-effectiveness. Additionally, the model computes incremental cost-effectiveness ratios (ICERs), which are calculated by dividing the cost variation by the variation in the proper measure of effectiveness.

### Sensitivity analysis

A one-way sensitivity analysis (OWSA) was conducted by using sequential bifurcation, a process that iteratively samples inputs and assesses the impact of each input against a pre-determined cost threshold value, to determine what variables affecting total treatment costs warrant focus during sensitivity analyses. Additionally, to test the robustness of the model concerning uncertainty in model input parameters, a probabilistic sensitivity analysis (PSA) is performed using a second-order Monte Carlo simulation with 1000 iterations. Each key model parameter is given a theoretical probability distribution in this analysis. A random number generator is used to draw parameter values from each distribution, and these values are run through the model to generate a cost-effectiveness scatter plot.

## Results

### Clinical outcome

**Fig. 2:**
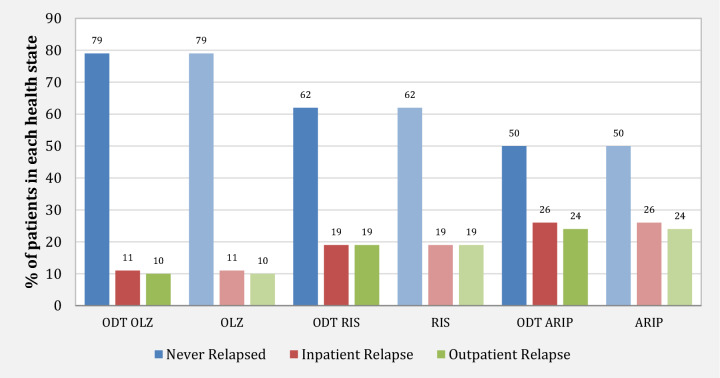
Relapse rates by treatment group. *ARIP* aripiprazole, *ODT* orally disintegrating tablet (formulation), *OLZ* olanzapine, *RIS* risperidone

The key clinical results for the base case are presented in Fig. 2. In general, the ODT and ODT + SOT as a group form of olanzapine were the most effective treatment option based on the study results. This combination resulted in the lowest percentages of outpatient relapse (10%) and inpatient relapse (11%), as well as the highest proportion of patients who remained stable and did not experience a relapse during the study period (79%). The study found that risperidone was the second most effective medication in terms of the clinical outcomes studied. However, the ODT and ODT + SOT as a group form of olanzapine resulted in the highest quality-adjusted life years (QALYs). The study results suggest that all three antipsychotic medications in ODT dosing form—olanzapine, risperidone, and aripiprazole—performed better than their respective SOT dosing form.

### Economic outcome

**Fig. 3:**
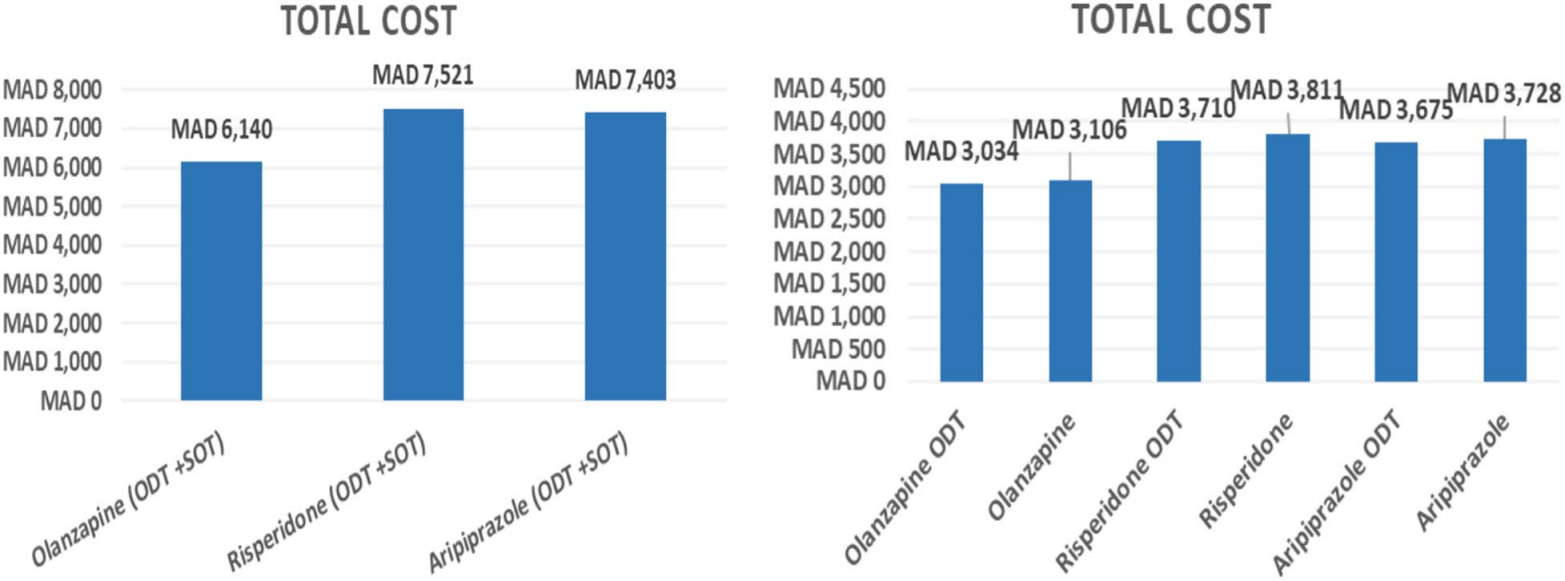
Base case economic cost

Figure 3 displays the direct healthcare costs for each treatment group in the base case. The model predicted that the mean total annual costs associated with olanzapine (ODT + SOT) and olanzapine ODT were the lowest (MAD 6140 and MAD 3034), with aripiprazole (ODT + SOT) and aripiprazole ODT having the second lowest estimated total direct medical cost (MAD 7403 and 3675) followed by risperidone (ODT + SOT) and risperidone ODT (MAD 7521 and 3710).

### Cost-effectiveness

The cost-effectiveness results for the base case are presented in Table 8. The results indicate that compared to the combined group of ODT + SOT forms of risperidone and aripiprazole, respectively, olanzapine (ODT + SOT) group therapy was more effective in terms of better QALYs and lesser direct costs. Table 8 provides direct comparisons between olanzapine (ODT + SOT) therapy and other treatment options. The findings suggest that olanzapine (ODT + SOT) therapy was cost-effective when compared to the combined group of ODT + SOT forms of risperidone (ICER: MAD 1,03,907), and ODT + SOT forms of aripiprazole (ICER: MAD 65,047).

**Table 8:** Base case cost-effectiveness results [intervention: olanzapine (ODT + SOT)]

Strategy	Total cost (MAD)	Total QALY	ICER (cost/QALY)
Olanzapine (ODT + SOT)	MAD 6140	1.5650	–
Risperidone (ODT + SOT)	MAD 7521	1.5517	− MAD 103,907
Aripiprazole (ODT + SOT)	MAD 7403	1.5456	− MAD 65,047

*ODT* orally disintegrating tablet (formulation), *QALY* quality-adjusted life years, *SOT* standard oral tablet [willingness to pay (WTP) = MAD 250,832.40]

The findings of the cost-effectiveness analysis for the base case (Table 9) indicate that using olanzapine ODT therapy instead of olanzapine SOT therapy resulted in lower costs (MAD 3034 compared to MAD 3106) and better health outcomes, as measured by the QALYs metric (0.7916 compared to 0.7733). As per Table 9, when directly comparing olanzapine ODT to other therapies, olanzapine ODT was found to be cost-effective compared to olanzapine SOT with an ICER of MAD 3921, risperidone ODT with an ICER of MAD 1,02,298, risperidone SOT with an ICER of MAD 31,088, and aripiprazole ODT or SOT formulations.

**Table 9:** Base case cost-effectiveness results (intervention: olanzapine ODT)

Strategy	Total cost (MAD)	Total QALY	ICER (cost/QALY)
Olanzapine ODT	MAD 3034	0.7916	–
Olanzapine	MAD 3106	0.7733	− MAD 3921
Risperidone ODT	MAD 3710	0.7850	− MAD 102,298
Risperidone	MAD 3811	0.7666	− MAD 31,088
Aripiprazole ODT	MAD 3675	0.7822	− MAD 68,032
Aripiprazole	MAD 3728	0.7633	− MAD 24,516

*ODT* orally disintegrating tablet (formulation), *QALY* quality-adjusted life years [willingness to pay (WTP) = MAD 250,832.40]

The relapse rates per treatment group are presented in Table 10, which suggests that the cost-effectiveness of both olanzapine ODT and olanzapine as a group (including ODT and SOT) is mainly influenced by their reduced percentages of relapse and a greater proportion of patients those remain stable.

**Table 10:** Base case relapse rates

	Proportion of stable patients (never relapsed) (%)	Relapse resulting in hospitalization (%)	Relapse resulting in an ambulatory visit (%)
Olanzapine	79	11	10
Risperidone	62	19	19
Aripiprazole	50	26	24
ODT olanzapine	79	11	10
ODT risperidone	62	19	19
ODT aripiprazole	50	26	24

### Sensitivity analyses results

**Fig. 4:**
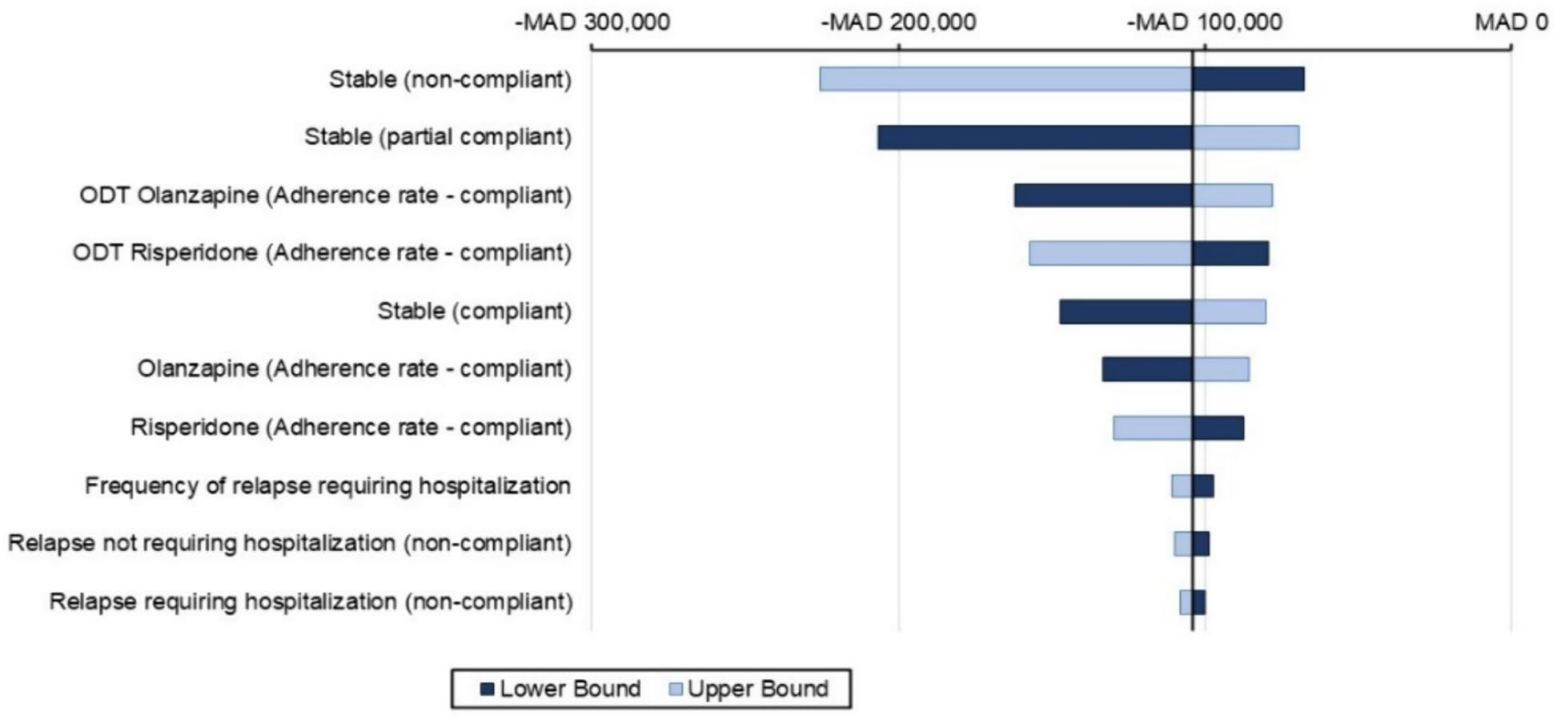
Tornado diagram for the ICER of olanzapine (ODT + SOT) vs. risperidone (ODT + SOT)

**Fig. 5:**
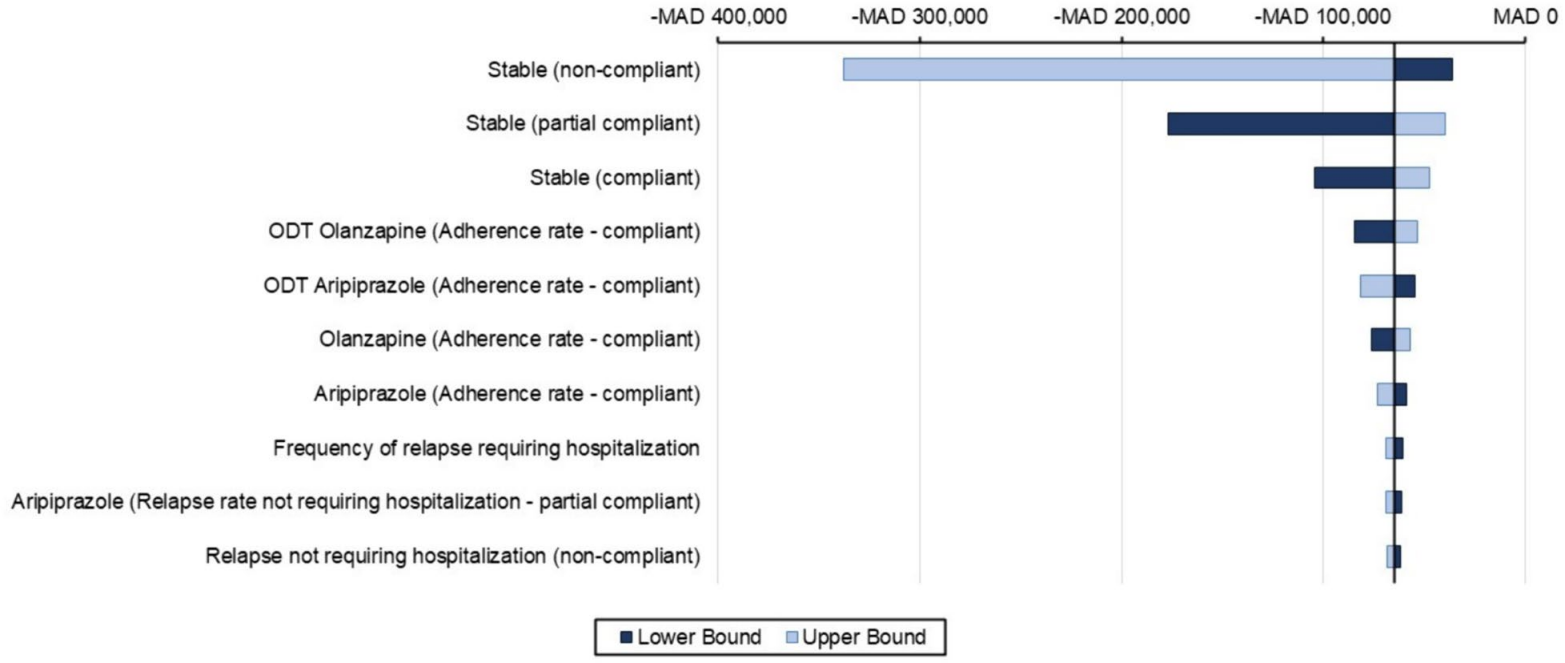
Tornado diagram for the ICER of olanzapine (ODT + SOT) vs. aripiprazole (ODT + SOT)

**Fig. 6:**
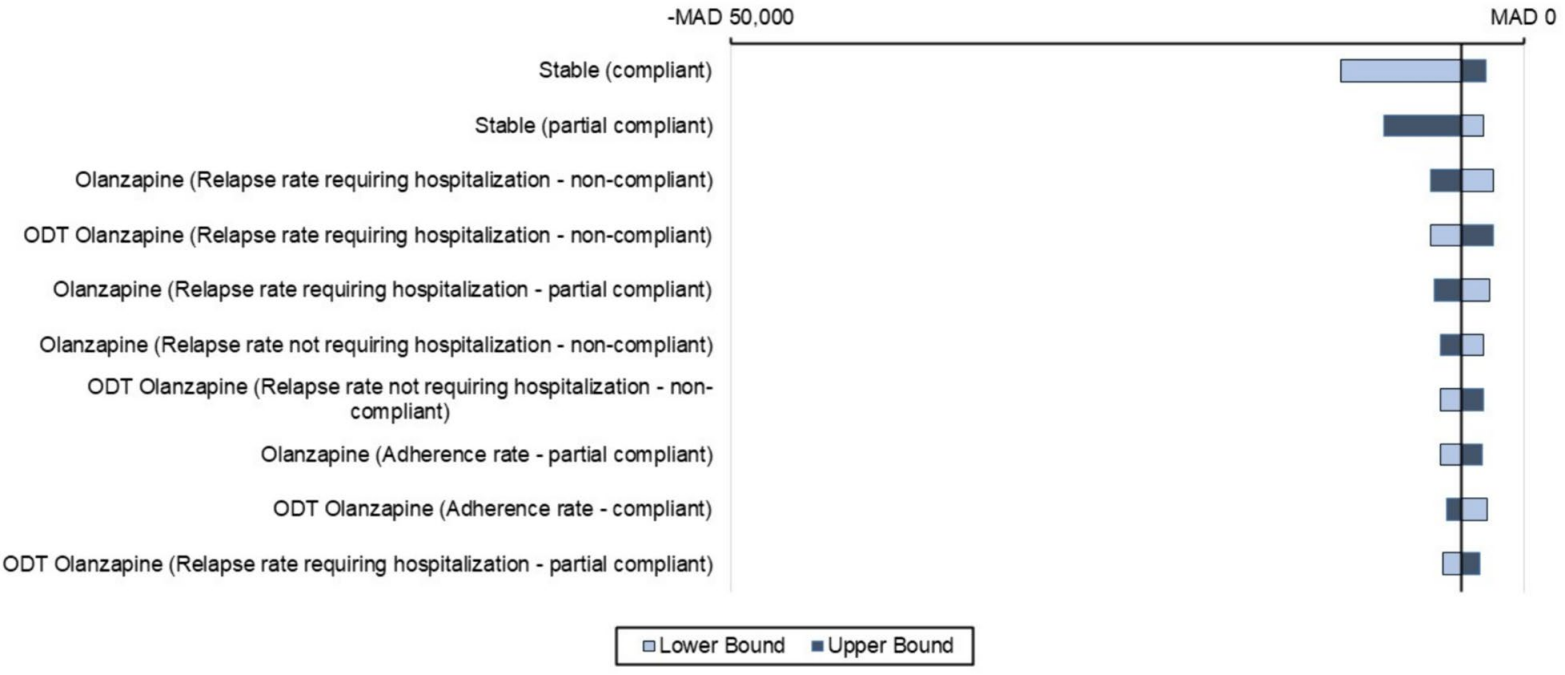
Tornado diagram for the ICER of olanzapine ODT vs. olanzapine SOT

**Fig. 7:**
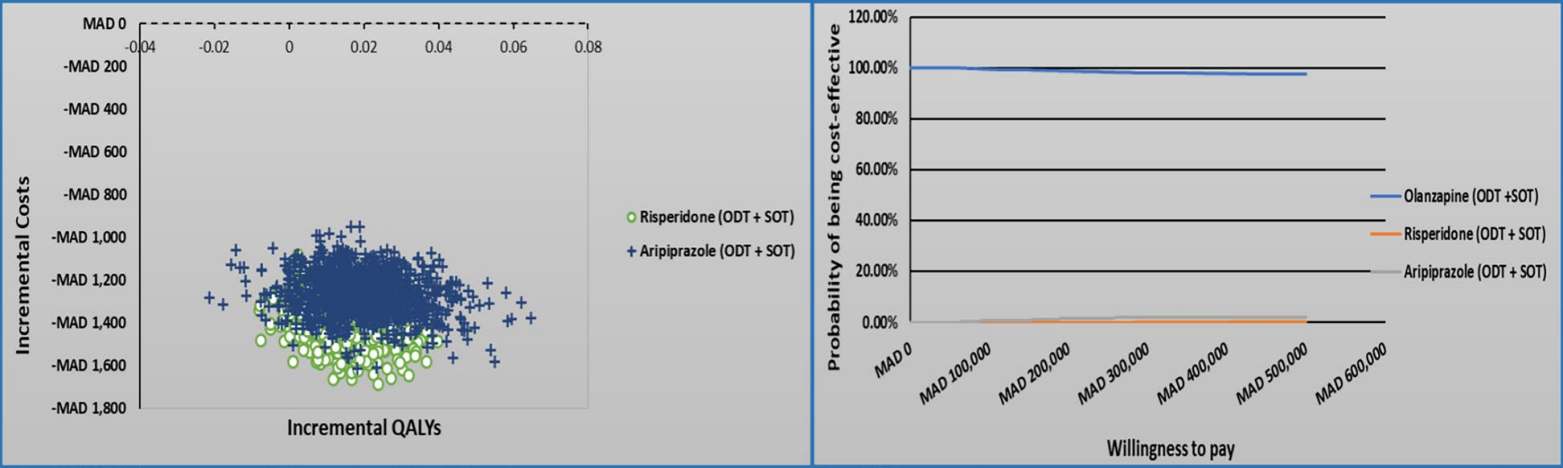
The cost-effectiveness plane and acceptability curves (ODT + SOT)

**Fig. 8:**
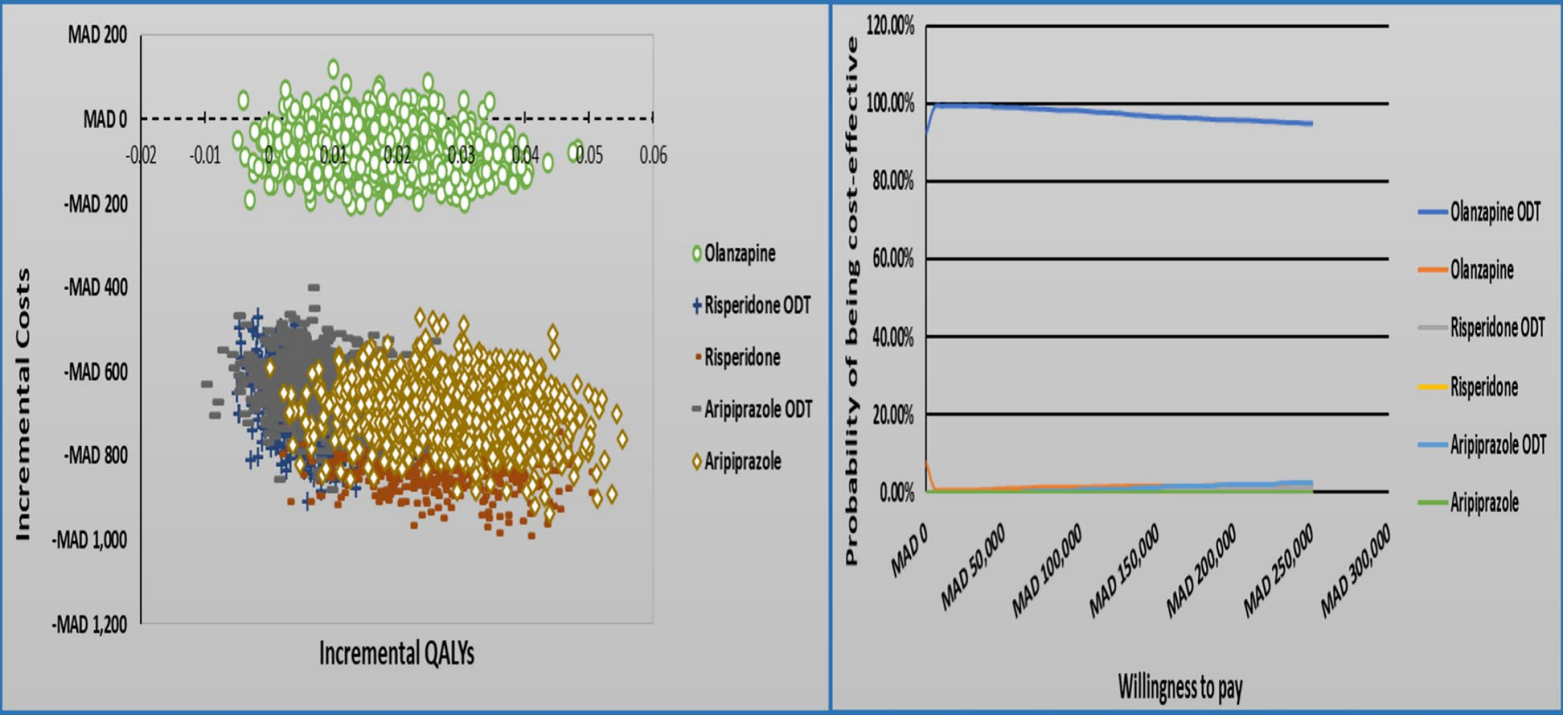
The cost-effectiveness plane and acceptability curves (ODT vs SOT)

1. One-way sensitivity analyses (OWSA)
  Figures 5, 6, and 7 presents a tornado diagram that illustrates how changes in individual parameter values affect the ICER, with a focus on the parameters that have the greatest impact on the ICER within a 1-year time horizon.
  - Olanzapine (ODT + SOT) vs. risperidone (ODT + SOT)
    The main parameter that had a significant impact on the cost-effectiveness comparison between olanzapine (ODT + SOT) and risperidone (ODT + SOT) is depicted in Fig. 4. It was observed that the utility value when the patient’s condition is stable under non-compliance medication with lower and upper ICER − MAD 67,491 and − MAD 225,671 was the most significant parameter among all the parameters measured. The second most significant parameter was the utility value when the patient’s condition was stable under partial compliance medication.
  - Olanzapine (ODT + SOT) vs. aripiprazole (ODT + SOT)
    Figure 5 displays the most important parameter that had a significant effect on the cost-effectiveness comparison between olanzapine (ODT + SOT) and aripiprazole (ODT + SOT). It was observed that the utility value of the patient’s stable condition under non-compliance medication with lower and upper ICER − MAD 35,985 and − MAD 338,119 was the most significant parameter among all the parameters measured. The second most significant parameter was the utility value when the patient’s condition was stable under partial compliance.
  - Olanzapine ODT vs. olanzapine SOT
    The main parameter that was found to have a significant impact on the cost-effectiveness comparison between olanzapine ODT and olanzapine SOT is illustrated in Fig. 6, which is the utility value of stable condition when taking medication as directed with lower and upper ICER − MAD 11,559 − MAD 2361 followed by the utility value of stable condition under partial compliance.
2. Probabilistic sensitivity analyses (PSA)
  In Fig. 7, a comparison of the costs and QALYs of olanzapine (ODT + SOT) vs. risperidone (ODT + SOT), and aripiprazole (ODT + SOT) is presented through a cost-effectiveness plane. The plot also includes the acceptability curves for these medications. At willingness to pay threshold of MAD 250832.40, there is an almost 100% likelihood that olanzapine (ODT + SOT) will be considered cost-effective.
  In Fig. 8, a comparison of the costs and QALYs of olanzapine ODT vs. olanzapine SOT, risperidone ODT and SOT, aripiprazole ODT and SOT is presented through a cost-effectiveness plane. The plot also includes the acceptability curves for these medications. At willingness to pay threshold of MAD 250832.40, the likelihood of olanzapine ODT to be cost-effective is greater than 90%.

## Discussion

This is the first study to be conducted for Morocco that measures the cost effectiveness of a novel drug formulation in the therapeutic management of schizophrenia by analyzing the economic feasibility of an ODT with its equivalent SOT formulation. Additionally, risperidone and aripiprazole, two other atypical antipsychotics that are also readily accessible in ODT and SOT formulations, were investigated alongside olanzapine ODT. Direct comparisons between olanzapine ODT and other treatments revealed that olanzapine ODT was more cost effective than olanzapine SOT with an ICER of MAD 3921, risperidone ODT with an ICER of MAD 1,02,298, risperidone SOT with an ICER of MAD 31,088, and formulations of aripiprazole ODT or SOT. In order to optimize the credibility and accountability of the model, we implemented one-way sensitivity analysis and probable sensitivity analysis since the premise for the model was variable compliance, persistence, and instances of relapse to examine the model’s ambiguity and the stability of the results and demonstrate the robustness of the base case findings. This was done in order to optimize the validity of the model and accountability.

The strength of our study stems from the way that the model was adapted from the US scenario and broadly addressed the same outcomes. Nonetheless, there is a significant difference between our model and the preceding model. Previously published studies only made individual comparisons between the olanzapine ODT group and the olanzapine SOT formulations and other treatment alternatives presented in ODT and SOT preparations [22, 29]. We have compared the combination of both ODT and SOT formulations for each treatment group. One of the limitations of this approach is that the findings of the model are limited to branded medications only and not applicable to any generic medications that may be available in the market.

A fundamental tenet of the model was that higher ODT adherence would result in better clinical outcomes, such as a reduced likelihood of relapse and hospitalization, and tailor the cost-effectiveness ratio. Olanzapine ODT has been linked to improved patient dispositions towards medication and improved medication adherence in both inpatient and outpatient settings, according to other studies [21, 30]. The results of our cost-effectiveness analysis are consistent with a recent cost-effectiveness study comparing olanzapine and aripiprazole in SOT formulations. [29] Although adherence had a substantial impact on the range of outcomes, relapse necessitating inpatient hospitalization—the most expensive aspect of schizophrenia treatment—was the primary driver of the model’s findings. A relationship between adherence and relapse, however, may exist since increased adherence is associated with a reduced probability of mental hospitalization in schizophrenia assistance [31, 32].

One of the primary shortcomings of this study was that it focused exclusively on acute therapy (successfully treated/relapse rate), drug costs alone, or a simple model architecture. The first economic analysis of olanzapine in both ODT and SOT formulations is provided to the decision-makers by this study. Second the study does not include parenteral formulations like long term injectables (LAIs) while evaluating the cost effectiveness of oral formulations against olanzapine ODT. Given that the primary objective of this research was to assess the cost-effectiveness of ODT formulations against the SOT formulation of olanzapine, LAIs were not considered for this study. Also, there is limited to no published literature available for Morocco, all data were sourced from peer-reviewed literature and from a clinical expert panel composed of experienced psychiatrists in Morocco. Another drawback in our study was due to the fact that our work was an adaptation of a previously published model, it is only instinctual that the original model’s drawbacks still apply. For example, some model input parameters (such as QALYs by health states) lack published medical literature, the model has a 1-year time horizon even though schizophrenia has a life-long course, and only direct medical expenses are taken into account. Lastly, we recognize that our model is adapted from a previously published one, we have validated it using scientifically accepted methods with data specific to Morocco. Our research aimed to develop a model reflecting the Moroccan psychiatric landscape providing a practical tool for decision/policy makers. To ensure that the model is tailored as per the local context, our study included comprehensive data collection from Moroccan psychiatrists and healthcare facilities. This collaboration ensured that the model appropriately represents Moroccan-specific clinical procedures and patient demographics. The participation of regional practitioners strengthens the contextual relevance of our findings.

## Conclusions

Results based on the model demonstrates that the use of an antipsychotic medication in its ODT formulation is more cost-effective than using its SOT formulation in the treatment of schizophrenia. More precisely, olanzapine ODT and olanzapine as a group (ODT + SOT) was found to be more cost-effective than olanzapine SOT, risperidone and aripiprazole in either ODT or SOT formulations or as a group (ODT + SOT). The model simulates real-world treatment processes and provides projections that should be used to inform decision-making processes from the Morocco healthcare system perspective. However, the findings may require further validation when local scientific data on relevant parameters becomes available for Morocco.

## Data Availability

All data generated or analysed during this study are included in this published article. No datasets were generated or analysed during the current study.

## Abbreviations

ARIP: Aripiprazole
DALYs: Disability adjusted life years
EPS: Extrapyramidal symptoms
ICER: Incremental cost-effectiveness ratio
MAD: Moroccan Dirham
MPR: Medication possession ratio
ODT: Orally dispersible tablets
OLZ: Olanzapine
OWSA: One way sensitivity analyses
PSA: Probabilistic sensitivity analysis
QALYs: Quality-adjusted life years
RIS: Risperidone
SOT: Standard oral tablet
WTP: Willingness to pay
YLDs: Years of healthy life lost due to disability

## References

[1] Schizophrenia. World Health Organization. 2022.

[2] Mental health: schizophrenia. The Scottish Public Health Observatory. 2023.

[3] Awad AG, Voruganti LNP. The burden of schizophrenia on caregivers. Pharmacoeconomics. 2008;26(2):149–62. 10.2165/00019053-200826020-00005. 18198934 10.2165/00019053-200826020-00005

[4] García-Ruiz AJ, Pérez-Costillas L, Montesinos AC, Alcalde J, Oyagüez I, Casado MA. Cost-effectiveness analysis of antipsychotics in reducing schizophrenia relapses. Health Econ Rev. 2012;2(1):8. 10.1186/2191-1991-2-8. 22828390 10.1186/2191-1991-2-8 PMC3402933

[5] Gee L, Pearce E, Jackson M. Quality of life in schizophrenia: a grounded theory approach. Health Qual Life Outcomes. 2003;1:31. 10.1186/1477-7525-1-31. 12952542 10.1186/1477-7525-1-31 PMC194222

[6] Knapp M, Mangalore R, Simon J. The global costs of schizophrenia. Schizophr Bull. 2004;30(2):279–93. 10.1093/oxfordjournals.schbul.a007078. 15279046 10.1093/oxfordjournals.schbul.a007078

[7] Murray CJL, Lopez AD. The Global burden of disease: a comprehensive assessment of mortality and disability from diseases, injuries, and risk factors in 1990 and projected to 2020. Cambridge: Harvard University Press; 1996.

[8] Turner JR, Wit M, Hajos T, Wit M, Howren MB, Insana S, Simonson MA. Quality-adjusted life years (QALYs). In: Encyclopedia of behavioral medicine. New York: Springer; 2013. p. 1605–6.

[9] Prieto L, Sacristán JA. Problems and solutions in calculating quality-adjusted life years (QALYs). Health Qual Life Outcomes. 2003;1:80. 10.1186/1477-7525-1-80. 14687421 10.1186/1477-7525-1-80 PMC317370

[10] Sanders GD, Neumann PJ, Basu A, Brock DW, Feeny D, Krahn M, Kuntz KM, Meltzer DO, Owens DK, Prosser LA, Salomon JA, Sculpher MJ, Trikalinos TA, Russell LB, Siegel JE, Ganiats TG. Recommendations for conduct, methodological practices, and reporting of cost-effectiveness analyses. JAMA. 2016;316(10):1093. 10.1001/jama.2016.12195. 27623463 10.1001/jama.2016.12195

[11] Drummond ME, Sculpher MJ, Torrance GW, O’Brien BJ, Stoddart GL. Methods for the economic evaluation of health care programmes. Oxford: Oxford University Press; 2005.

[12] Mathers CD, Vos ET, Stevenson CE, Begg SJ. The burden of disease and injury in Australia. Bull World Health Organ. 2001;79(11):1076–84. 11731817 PMC2566696

[13] Velligan DI, Rao S. The epidemiology and global burden of schizophrenia. J Clin Psychiatry. 2023;84(1):45094. 10.4088/JCP.MS21078COM5. 10.4088/JCP.MS21078COM5 36652681

[14] Charlson FJ, Ferrari AJ, Santomauro DF, Diminic S, Stockings E, Scott JG, McGrath JJ, Whiteford HA. Global epidemiology and burden of schizophrenia: findings from the global burden of disease study 2016. Schizophr Bull. 2018;44(6):1195–203. 10.1093/schbul/sby058. 29762765 10.1093/schbul/sby058 PMC6192504

[15] Greene MC, Yangchen T, Lehner T, Sullivan PF, Pato CN, McIntosh A, Walters J, Gouveia LC, Msefula CL, Fumo W, Sheikh TL, Stockton MA, Wainberg ML, Weissman MM. The epidemiology of psychiatric disorders in Africa: a scoping review. Lancet Psychiatry. 2021;8(8):717–31. 10.1016/S2215-0366(21)00009-2. 34115983 10.1016/S2215-0366(21)00009-2 PMC9113063

[16] Jörns-Presentati A, Napp A-K, Dessauvagie AS, Stein DJ, Jonker D, Breet E, Charles W, Swart RL, Lahti M, Suliman S, Jansen R, van den Heuvel LL, Seedat S, Groen G. The prevalence of mental health problems in sub-Saharan adolescents: a systematic review. PLoS ONE. 2021;16(5): e0251689. 10.1371/journal.pone.0251689. 33989357 10.1371/journal.pone.0251689 PMC8121357

[17] Rate of disease burden from schizophrenia: 1990 to 2019. 2019.

[18] Lehman AF, Lieberman JA, Dixon LB, McGlashan TH, Miller AL, Perkins DO, Kreyenbuhl J, American Psychiatric Association, & Steering Committee on Practice Guidelines. Practice guideline for the treatment of patients with schizophrenia, second edition. Am J Psychiatry. 2004;161(2 Suppl):1–56. 15000267

[19] Ginovart N, Kapur S. Role of dopamine D2 receptors for antipsychotic activity. Handbook of experimental pharmacology, 2012;27–52. 10.1007/978-3-642-25761-2_2 10.1007/978-3-642-25761-2_2 23129327

[20] Narasimhan M, Bruce TO, Masand P. Review of olanzapine in the management of bipolar disorders. Neuropsychiatr Dis Treat. 2007;3(5):579–87. 19300587 PMC2656294

[21] Czekalla J, Wagner T, Schacht A, Kluge M, Kinon B. Effectiveness and medication acceptance of olanzapine disintegrating tablets compared to standard olanzapine tablets in acutely treated psychiatric patients. Patient Prefer Adherence. 2007;1:19–27. 10.2147/ppa.s2300. 19956444 10.2147/ppa.s2300 PMC2779125

[22] Ascher-Svanum H, Furiak NM, Lawson AH, Klein TM, Smolen LJ, Conley RR, Culler SD. Cost-effectiveness of several atypical antipsychotics in orally disintegrating tablets compared with standard oral tablets in the treatment of schizophrenia in the United States. J Med Econ. 2012;15(3):531–47. 10.3111/13696998.2012.662923. 22304338 10.3111/13696998.2012.662923

[23] Haddad P, Brain C, Scott J. Nonadherence with antipsychotic medication in schizophrenia: challenges and management strategies. Patient Relat Outcome Meas. 2014;5:43. 10.2147/PROM.S42735. 25061342 10.2147/PROM.S42735 PMC4085309

[24] Furiak NM, Ascher-Svanum H, Klein RW, Smolen LJ, Lawson AH, Montgomery W, Conley RR. Cost-effectiveness of olanzapine long-acting injection in the treatment of patients with schizophrenia in the United States: a micro-simulation economic decision model. Curr Med Res Opin. 2011;27(4):713–30. 10.1185/03007995.2011.554533. 21265593 10.1185/03007995.2011.554533

[25] Chong HY, Teoh SL, Wu DB-C, Kotirum S, Chiou C-F, Chaiyakunapruk N. Global economic burden of schizophrenia: a systematic review. Neuropsychiatr Dis Treat. 2016;12:357–73. 10.2147/NDT.S96649. 26937191 10.2147/NDT.S96649 PMC4762470

[26] Juma K, Wekesah FM, Kabiru CW, Izugbara CO. Burden, drivers, and impacts of poor mental health in young people of West and Central Africa: implications for research and programming. In: West African youth challenges and opportunity pathways. Cham: Springer International Publishing; 2020. p. 233–65. 10.1007/978-3-030-21092-2_11.

[27] Fabrazzo M, Cipolla S, Camerlengo A, Perris F, Catapano F. Second-generation antipsychotics’ effectiveness and tolerability: a review of real-world studies in patients with schizophrenia and related disorders. J Clin Med. 2022;11(15):4530. 10.3390/jcm11154530. 35956145 10.3390/jcm11154530 PMC9369504

[28] Novick D, Montgomery W, Treuer T, Koyanagi A, Aguado J, Kraemer S, Haro JM. Comparison of clinical outcomes with orodispersible versus standard oral olanzapine tablets in nonadherent patients with schizophrenia or bipolar disorder. Patient Prefer Adherence. 2017;11:1019–25. 10.2147/PPA.S124581. 28652711 10.2147/PPA.S124581 PMC5476712

[29] Zhao J, Jiang K, Li Q, Zhang Y, Cheng Y, Lin Z, Xuan J. Cost-effectiveness of olanzapine in the first-line treatment of schizophrenia in China. J Med Econ. 2019;22(5):439–46. 10.1080/13696998.2019.1580714. 30732487 10.1080/13696998.2019.1580714

[30] Kinon BJ, Hill AL, Liu H, Kollack-Walker S. Olanzapine orally disintegrating tablets in the treatment of acutely ill non-compliant patients with schizophrenia. Int J Neuropsychopharmacol. 2003;6(2):S1461145703003389. 10.1017/S1461145703003389. 10.1017/S1461145703003389 12890301

[31] Law MR, Soumerai SB, Ross-Degnan D, Adams AS. A longitudinal study of medication nonadherence and hospitalization risk in schizophrenia. J Clin Psychiatry. 2008;69(1):47–53. 10.4088/JCP.v69n0107. 18312037 10.4088/jcp.v69n0107

[32] Valenstein M, Copeland LA, Blow FC, McCarthy JF, Zeber JE, Gillon L, Bingham CR, Stavenger T. Pharmacy data identify poorly adherent patients with schizophrenia at increased risk for admission. Med Care. 2002;40(8):630–9. 10.1097/00005650-200208000-00002. 12187177 10.1097/00005650-200208000-00002

